# Bridging Gaps in Hepatitis Delta Virus Care: A Cross-Sectional Mixed-Methods Study of Clinician Perspectives From Major Urban Centers in Pakistan

**DOI:** 10.7759/cureus.99722

**Published:** 2025-12-20

**Authors:** Saeed Hamid, Syed Munawar Ali, Zaigham Abbas, Ghias Un Nabi Tayyab, Rao Saad Ali Khan, Asher Bin Feroze, Saad K Niaz

**Affiliations:** 1 Internal Medicine, Aga Khan University, Karachi, PAK; 2 Clinical Operations, IQVIA, Karachi, PAK; 3 Gastroenterology and Hepatology, Dr. Ziauddin University Hospital, Karachi, PAK; 4 Gastroenterology and Hepatology, Doctors Hospital & Medical Center, Lahore, PAK; 5 Gastroenterology, Pak-Emirates Military Hospital, Rawalpindi, PAK; 6 Statistics, Statistical Processing and Data Analytics Expert, Karachi, PAK; 7 Gastroenterology and Hepatology, Sindh Institute of Advanced Endoscopy and Gastroenterology/Dow University of Health Sciences (DUHS), Karachi, PAK

**Keywords:** clinicians’ perspectives, diagnosis, emergency use authorization, hepatitis delta virus, pakistan, treatment access

## Abstract

Background

Hepatitis delta virus (HDV) is the most aggressive form of viral hepatitis, accelerating progression to cirrhosis, hepatocellular carcinoma, and liver failure. Despite its severity, HDV remains underdiagnosed and under-prioritized in Pakistan, where hepatitis B virus (HBV) prevalence is high. Understanding clinician perspectives is essential for informing national strategies related to diagnosis, treatment, and policy integration.

Methods

A non-interventional, cross-sectional mixed-methods survey was conducted among 12 clinicians, including hepatologists, gastroenterologists, and infectious disease specialists from Karachi, Islamabad/Rawalpindi, Lahore, Peshawar, and Nowshera. Data were collected using a structured questionnaire adapted from international HDV surveys and contextualized for Pakistan. Quantitative data were analyzed descriptively, while qualitative responses underwent thematic analysis to identify barriers, gaps, and recommendations.

Results

Twelve clinicians from major urban centers in Pakistan participated, representing diverse specialties with 5-30+ years of experience. Fifty percent reported frequently encountering HDV among HBV patients, and most estimated HDV co-infection rates between 10% and 20%. Anti-HDV testing was routinely performed by 75% of clinicians, whereas HDV RNA PCR was routinely performed by only 50%, primarily due to high cost and limited availability; 83.3% reported that HDV diagnostics were available but unaffordable for most patients. Key diagnostic barriers included high cost (75%), lack of awareness (66.7%), limited test availability (41.7%), and absence of national screening guidelines (50%). Two-thirds classified HDV severity as moderate, while 33.4% rated it severe or life-threatening. Perceived clinical risks included rapid progression to cirrhosis (75%), liver failure (66.7%), hepatocellular carcinoma (66.7%), and increased mortality (33.3%). Most clinicians identified major treatment gaps, with 75-83.3% describing an urgent or critical need for a national HDV treatment program. Policy support was strong: 83.3% favored HDV inclusion in the National Hepatitis Program, 83.3% endorsed national screening guidelines and awareness campaigns, and all clinicians (100%) supported an Emergency Use Authorization pathway with safeguards such as Phase II/III data and advisory oversight. Qualitative insights reinforced concerns about worsening liver disease burden, inequitable access, and escalating healthcare costs without immediate policy action.

Conclusion

This study reveals substantial diagnostic, therapeutic, and policy gaps in HDV management in Pakistan. Clinicians strongly advocate for nationwide screening, cost reduction of diagnostics, integration of HDV into the national hepatitis program, and early treatment access through regulatory innovation. Urgent action is needed to prevent worsening disease burden, improve equity, and align with global hepatitis elimination targets.

## Introduction

Hepatitis delta virus (HDV) is a defective RNA virus that depends on hepatitis B virus (HBV) for entry, replication, and virion assembly [1]. Based on pooled global estimates, HDV prevalence is approximately 0.8% in the general population and 13% among HBV carriers, corresponding to an estimated 48-60 million infections worldwide [1]. HDV is clinically significant because of its rapid progression; cirrhosis can develop within five years, and hepatocellular carcinoma within a decade of infection [2].

South Asia, particularly Pakistan, is a region of high endemicity. Local research approximates the prevalence of HDV among HBV-positive patients to be between 20% and 35% [3]. The most common is genotype 1, whereby the young age groups are disproportionately affected; co-infections are prevalent among people below the age of 40 years [4]. There is some evidence of HDV modifying the dynamics of HBV, where some studies have indicated that HBV replication is suppressed in dual infections [5]. These results highlight the complexity of HDV and its role in the burden of chronic liver disease in Pakistan, a nation with an existing high burden of viral hepatitis [6].

Although HDV has clinical and public health implications, it is an overlooked aspect of hepatitis policy in Pakistan. Diagnostic capacity is not constant, and RNA PCR testing is restricted and unaffordable to patients. There are also gaps in provider knowledge that hinder care since most clinicians are unsure of screening criteria or treatment routes [7,8]. These issues are exacerbated by structural harms like unsafe medical practices, socioeconomic disparities, and the absence of national guidelines [9-11].

Considering the high incidence and poor prognosis of HDV, there is an imminent necessity to enhance diagnostic practices, increase access to treatment, and make HDV a part of the national campaign to eliminate hepatitis. Nevertheless, the clinical perspective on HDV in Pakistan has not been explored extensively, even though it is at the core of defining screening policy, treatment regimens, and regulatory frameworks, including emergency use authorization (EUA). To fill this gap, the current study evaluated the experience of Pakistani clinicians with the diagnosis of HDV, difficulties with treatment, and the perceived burden of disease. It also investigated the advice of experts on how the policy, screening, and access to treatment could be enhanced, with the aim of producing evidence to inform the integration of HDV into the national hepatitis program.

## Materials and methods

Study design and setting

This is a mixed-method, non-interventional, and cross-sectional survey (quantitative and qualitative components) that was carried out in major urban areas in Pakistan, namely Karachi, Lahore, Islamabad/Rawalpindi, Peshawar, and Nowshera, from September to November 2025. Major urban centers were purposefully selected because they host the main tertiary hepatology and infectious disease centers and are hubs for complex HBV/HDV care and policy influence. This purposive, urban-centered sampling was chosen to capture expert opinion from clinicians most involved in HDV management, but it introduces an urban-centric bias that limits generalizability to rural and primary-care settings.

Ethics

Ethical exemption was obtained from the National Bioethics Committee for Research (NBC-R) of Pakistan prior to study initiation (Ref: No.4-87/NBCR-1312/25-26/239; Date 5th August 2025). Participation was voluntary, and electronic informed consent was required before enrollment. Confidentiality was ensured through anonymized survey responses, encrypted storage of datasets, and restricted access to the principal investigator and designated team members. The study involved no interventions and did not pose any physical, psychological, or social risks. All procedures were conducted in accordance with the Declaration of Helsinki, Good Clinical Practice (GCP) guidelines, and World Health Organization standards for non-interventional survey research.

Participants

Clinicians were recruited through purposive sampling using email invitations and professional networks, including national hepatology and gastroenterology societies. Eligible participants included gastroenterologists, hepatologists, and infectious disease/internal medicine clinicians who actively manage HBV and HDV patients; overlapping roles were treated as functional rather than distinct categories. Inclusion required at least five years of clinical experience in a recognized public or private tertiary-care institution. Clinicians not currently managing HBV/HDV cases, those in primarily administrative roles, or those unwilling to provide consent were excluded. Most respondents practiced in major urban tertiary centers, with representation from both public and private sectors.

Variables

The primary variables of interest included clinicians’ practices and perspectives on HDV diagnosis (use of serological and molecular testing), perceived disease burden, therapeutic challenges, and barriers to effective care, such as limited access to diagnostics, regulatory hurdles, and financial constraints. Additional variables included clinician perceptions of HDV severity, clinical consequences of untreated or undiagnosed infection, urgency of a national-level treatment program, and awareness and attitudes toward investigational therapies and emergency use authorization (EUA) pathways.

Data sources and measurement

Data were collected using a Microsoft Forms-based questionnaire adapted from international HDV survey tools, ensuring consistency with global instruments and increasing transparency of the study design. The instrument contained both quantitative data (e.g., on diagnostic barriers, disease severity, treatment access, and policy measures) and open-ended items to elicit qualitative insights. Prior to full deployment, the questionnaire was pilot tested among two hepatologists to refine clarity, reliability, and content validity.

Study size

The final sample consisted of 12 expert clinicians. Although modest, this sample was appropriate for an exploratory study targeting a highly specialized population. Given that HDV specialists in Pakistan represent a small and concentrated expert group, the sample size aligns with WHO recommendations for expert-opinion surveys, which prioritize consultation with a limited number of qualified subject-matter experts rather than large numerical samples. This approach is consistent with WHO guidance for non-interventional, policy-informing research [12]. The selected sample, therefore, provided sufficient depth of insight for policy-oriented analysis.

Statistical methods

Quantitative data were analyzed using IBM Corp. Released 2019. IBM SPSS Statistics for Windows, Version 25. Armonk, NY: IBM Corp. and Microsoft Excel. Descriptive statistics, including frequencies, percentages, means, and cross-tabulations, were used to summarize survey findings. Results were presented in tabular and graphical form to illustrate the distribution of responses across categories. Qualitative data were analyzed using reflexive thematic analysis following Braun and Clarke’s approach. Two reviewers independently generated initial codes, grouped them into candidate categories, and iteratively refined these into final themes. Any coding discrepancies were resolved through discussion and consensus; although a third reviewer was available, their input was ultimately not required. Given the exploratory nature of the study and the small sample size, no inferential statistical tests were performed. Instead, quantitative and qualitative findings were synthesized to develop evidence-based recommendations for clinical and policy action. Formal saturation metrics were not calculated due to the predefined expert sample, but thematic redundancy was observed, with similar concepts repeatedly emerging across respondents’ narratives.

## Results

A total of 12 clinicians participated in the study, representing diverse professional designations and geographic regions. Most respondents were based in Karachi, followed by Islamabad/Rawalpindi and Lahore, with additional representation from Noshera and Peshawar. Participants included professors, consultants, assistant professors, and coordinators, with clinical experience ranging from five years to over 30 years. The distribution of demographic characteristics is shown in Table 1.

**Table 1:** Demographics and General Information of Respondents (N = 12).

Variables		N	%
Designation	Professor / HOD	4	33.3
	Assistant Professor / Doctor	2	16.7
	Consultant	2	16.7
	Coordinator	1	8.3
	Not reported	3	25.0
City of Practice	Islamabad / Rawalpindi	3	25.0
	Karachi	5	41.7
	Lahore	2	16.7
	Nowshera	1	8.3
	Peshawar	1	8.3
Years of Experience	5–10 years	2	16.7
	11–20 years	4	33.3
	21–30 years	4	33.3
	31+ years	2	16.7

Half of the clinicians reported frequently encountering HDV-positive patients in their practice, while one-third encountered them occasionally. Respondents perceived that approximately 10-20% of their HBV patients were co-infected with HDV, while some reported lower estimates.

Diagnostic practices varied; 75% routinely performed anti-HDV (IgG or total), whereas only 50% performed HDV RNA testing. Affordability was identified as a major challenge, with most clinicians stating that diagnostic tests were available but unaffordable for patients. The main challenges cited were high costs, limited test availability, lack of awareness, and absence of national screening guidelines. Findings are detailed in Table 2.

**Table 2:** Diagnostic Practices and Perceived Disease Burden Among Clinicians (N = 12).

Variables		N	%
Frequency of Encounter HDV-positive Patients	Frequently	6	50.0%
	Occasionally	4	33.3%
	Rarely	1	8.3%
	Very frequently	1	8.3%
Estimated HBV patients co-infected with HDV	<5%	3	25.0%
	5–10%	3	25.0%
	10–20%	4	33.3%
	>20%	2	16.7%
Challenges in Diagnosing HDV	Lack of awareness	8	66.7%
	Limited testing availability	5	41.7%
	High cost of testing	9	75.0%
	Lack of national screening guidelines	6	50.0%
Frequency of Anti-HDV (IgG or total)	Rarely Performed	2	16.7%
	Occasionally Performed	1	8.3%
	Routinely Performed	9	75.0%
Frequency of HDV RNA testing	Rarely Performed	1	8.3%
	Occasionally Performed	4	33.3%
	Routinely Performed	6	50.0%
	Not Available	1	8.3%

Clinicians were asked to rate the severity of HDV and identify the consequences of delayed or absent treatment. Two-thirds classified HDV as “moderate,” while the remainder described it as severe or critically life-threatening. Respondents highlighted rapid progression to cirrhosis, increased risk of liver failure and hepatocellular carcinoma, poor response to HBV treatments, and higher mortality as major consequences (Table 3).

**Table 3:** Disease Severity & Public Health Risk. *Participants could select more than one option; percentages do not sum to 100%

Variables		N	%
Severity of HDV Compared to Other Viral Hepatitis	Mild	0	0.0
	Moderate	8	66.7
	Severe	2	16.7
	Critically Life-Threatening	2	16.7
Clinical Consequences of Undetected/Untreated HDV*	Rapid progression to cirrhosis	9	75.0%
	Higher risk of liver failure	8	66.7%
	Increased risk of hepatocellular carcinoma	8	66.7%
	Poor response to existing HBV treatments	6	50.0%
	Increased mortality	4	33.3%
	Others	1	8.3%

Clinicians underscored significant treatment gaps. When asked about barriers to HDV-specific care, they cited rapid progression to cirrhosis, high risk of liver failure, hepatocellular carcinoma, poor treatment response, and increased mortality. Urgency for a national-level treatment access program was widely recognized, with most rating it as either very urgent or critical (Table 4).

**Table 4:** Treatment Access & Infrastructure.

Variables		N	%
Affordability of HDV Diagnostic Tests	Affordable and widely accessible	2	16.7%
	Available but not affordable for most patients	10	83.3%
Barriers in Accessing HDV-specific Treatment	Rapid progression to cirrhosis	9	75.0%
	Higher risk of liver failure	8	66.7%
	Increased risk of hepatocellular carcinoma	8	66.7%
	Poor response to existing HBV treatments	6	50.0%
	Increased mortality	4	33.3%
Urgency of National HDV Treatment Program	Not Urgent	0	0.0%
	Moderately urgent	3	25.0%
	Very urgent	6	50.0%
	Critical and immediate	3	25.0%

Policy, treatment access, and EUA perspectives

When asked whether HDV should be added as a separate component of the National Hepatitis Program, 10 of 12 clinicians (83.3%) responded ‘yes,’ one (8.3%) responded ‘no,’ and one (8.3%) was ‘unsure.’ A strong consensus emerged on policy measures; most respondents recommended the formation of national HDV screening guidelines and widespread awareness campaigns to control HDV in Pakistan (Table 5).

**Table 5:** Strategies Recommended to Control HDV in Pakistan.

Variables	N	%
National HDV screening guidelines	10	83.30%
Awareness campaigns at community and physician level	10	83.30%
Public-private partnerships to introduce HDV drugs	3	25.00%
HDV-specific training for doctors and nurses	2	16.70%
International collaboration for access to investigational drugs	4	33.30%

Awareness of investigational and conditionally approved HDV drugs was high (83.3%). Importantly, all participants endorsed an EUA mechanism for Pakistan if supported by promising safety and efficacy data. Key safeguards included availability of Phase II or Phase III trial data and prior international approval, preferably supported by safety and efficacy evidence from pivotal trials involving Pakistani HDV patients, as well as oversight by a national advisory board and registry-based patient monitoring. Clinicians strongly supported establishing a DRAP task force and pilot access programs before full drug registration (Table 6).

**Table 6:** Emergency Use Authorization Perspectives (N = 12).

Variables		N	%
Awareness of investigational/conditionally approved HDV drugs	Yes	10	83.3
	No	2	16.7
Support for EUA in Pakistan	Yes	12	100.0%
	No	0	0.0%
Safeguards for Emergency Access	Availability of Phase II interim data, preferably supported by safety and efficacy evidence from pivotal trials involving Pakistani HDV patients	8	66.7%
	Drug is already approved in other countries or under compassionate use	10	83.3%
	High unmet medical need with no local alternatives	5	41.7%
	Oversight by national expert advisory board	7	58.3%
	Patient consent and registry-based monitoring	4	33.3%
DRAP Special Taskforce for Fast-track Review	Yes	10	83.3
	No	1	8.3
	Only if endorsed by WHO or regional health authorities	1	8.3
Support for Pilot Treatment Access Programs	Yes	9	75.0
	No	1	8.3
	Only with government oversight and sponsor responsibility	2	16.7

Qualitative findings

Consequences of Inaction

Respondents anticipated a worsening liver disease burden, including progression to cirrhosis, hepatocellular carcinoma, and liver failure (Table 7). Across the 12 clinicians, nine explicitly mentioned accelerated cirrhosis, eight reported increased risk of liver failure and HCC, and seven highlighted the likelihood of rising healthcare costs and financial strain. Several also noted the growing demand for liver transplantation, increased mortality, poor quality of life, and widening inequities in healthcare access. Missed elimination targets were frequently cited as a major systemic consequence.

**Table 7:** Thematic Synthesis of Expert Recommendations on National HDV Response.

Theme	Illustrative Insights from Experts	Representative Quote
Consequences if HDV is not addressed	Worsening disease burden with rapid progression to cirrhosis, HCC, liver failure; increased mortality; higher transplantation needs; widening inequities; escalating costs	“If we keep missing HDV, patients will continue presenting directly with cirrhosis.”
Recommended government actions	Nationwide HDV screening, epidemiological data collection, awareness campaigns, subsidized diagnostics, early access to bulevirtide, maternal vaccination	“Guidelines are needed, but RNA tests must be affordable or nothing will change.”
Models and precedents	EMA conditional approval, real-world evidence models, COVID-19 mechanisms, Punjab AIDS Program, WHO emergency frameworks	“Pakistan can adapt conditional approval models already used internationally.”

Recommended Government Actions

Participants emphasized the urgent need for nationwide HDV screening for all HBsAg-positive patients, supported by epidemiological data and standardized guidelines. Awareness and education campaigns at both the physician and community levels were repeatedly recommended. Other suggestions included improved access to bulevirtide, cost reduction for testing, integration of HDV into HBV vaccination, maternal and birth-dose vaccination, elimination of unregulated practices, and support for clinical trials through public-private partnerships.

Suggested Models and Precedents

Respondents cited both international and local models that could inform HDV policy in Pakistan. International precedents included EMA conditional approval frameworks, compassionate and expanded access programs, real-world evidence models, and strategies used during the COVID-19 response. Locally, participants highlighted the Punjab AIDS Control Program and proposed adapting the WHO’s Health Emergencies Programme and UNAIDS’ rights-based approaches. Industry-led initiatives such as Gilead’s HCV treatment access program were also noted.

## Discussion

This study provides important insights into clinicians’ perspectives on the burden, diagnosis, and policy needs of hepatitis delta virus (HDV) in Pakistan. Nearly half of respondents reported frequent encounters with HDV-positive patients, and most estimated co-infection rates between 10% and 20% among their hepatitis B virus (HBV) populations. These observations are consistent with national epidemiological studies showing a substantial burden of HDV [5]. A hospital-based study of 1,394 pregnant women in Lahore reported an HDV co-infection prevalence of 20.6% among HBV-positive women, although the overall anti-HDV prevalence in the full sample was much lower at 1.9% (95% CI -0.7 to 4.7) [9]. Consistently, studies in specific high-risk groups, such as those seeking hepatitis B vaccination in Karachi, have found much higher rates of 26.2% [10]. Globally, HDV remains the most aggressive form of viral hepatitis, progressing to cirrhosis and hepatocellular carcinoma more rapidly than HBV alone [11,13]. Interestingly, two-thirds of respondents classified HDV as a ‘moderate’ health threat, which contrasts with global data describing HDV as the most aggressive form of viral hepatitis [14,15]. This discrepancy may reflect perception bias; clinicians in tertiary centers frequently manage advanced cirrhosis and hepatocellular carcinoma, which may recalibrate their internal ‘severity’ scale and lead them to rate HDV as moderate relative to other catastrophic liver conditions they routinely encounter. In addition, many respondents practice in settings where only the most severe cases are referred, so ‘moderate’ may represent an average impression across a spectrum of disease. 

Pakistan’s HBV and HDV epidemiology is heterogeneous across provinces and populations. Higher HDV co-infection rates have been reported in Sindh and Balochistan and in specific high-risk groups, such as individuals attending hepatitis clinics, blood banks, or vaccination centers, whereas lower estimates come from community-based or antenatal cohorts. Many of these figures are derived from hospital-based convenience samples, small cross-sectional surveys, or regional registries rather than large national probability samples, which partly explains the variation in reported prevalence. Our findings, therefore, need to be interpreted as clinician impressions emerging from major urban tertiary centers, rather than precise estimates of provincial or national disease burden.

Significant gaps were identified in clinicians’ HDV diagnostic practices. Although the majority of the cases recorded the regular use of HDV antibody testing, molecular confirmation through RNA PCR was not regularly available and was commonly stated to be unaffordable to the patient. Besides these affordability issues, clinicians also specifically identified a lack of awareness and a lack of testing as barriers, as well as the lack of national screening guidelines as constraints on achieving the desired outcomes, and thus highlighted the importance of system-level changes, along with education at the provider level. This corresponds with previous research indicating that cost and access are significant impediments to HDV diagnosis in Pakistan [16]. International recommendations indicate the necessity of filling this gap; the EASL Clinical Practice Guidelines (2023) suggest performing systematic HDV antibody screening in all HBsAg-positive individuals with RNA PCR validation of antibody-positive individuals [17]. Similarly, the Chronic Liver Disease Foundation recommendations (2023) also introduce a standardized diagnostic algorithm that implies universal screening [18]. The clinicians’ concerns in this study, therefore, align with both the literature and international best practice, underscoring the need for national policies to improve access to affordable diagnostics.

Policy perspectives captured in this study were strikingly consistent. Most clinicians supported introducing HDV as an independent entity to the Pakistan National Hepatitis Program, the establishment of national screening strategies, and expenditure on physician and community education. Notably, a national-level HDV treatment program was also rated as very urgent or critical by most clinicians, indicating a high sense of urgency when it comes to closing the gap. Similar suggestions have been seen in other low- and middle-income countries (LMICs), in which no specific HDV guidelines have led to ineffective case finding and management [19]. Recent agreement-based recommendations of the Gulf Cooperation Council also echo the same by asserting that there is a need to have public awareness, training of the healthcare providers, and standardized testing procedures in order to enhance control of HDV in the endemic areas [17]. These results draw on a common regional understanding that national hepatitis plans should explicitly include HDV.

The other important outcome was that emergency use authorization (EUA) pathways of new therapies like bulevirtide were unanimously approved. Participants were also more aware of investigational and conditionally approved drugs, potentially one reason they showed such strong support for EUA mechanisms. This is indicative of clinician support for regulatory innovation in the context of a few treatment options. On a global scale, the European Medicines Agency (EMA) granted conditional approval to bulevirtide in 2020 with the help of real-world evidence frameworks and the broader access programs [20]. The accelerated pathways were commonly used during the COVID-19 pandemic and proved feasible within resource-limited conditions. Critical first steps, according to respondents in this study, are a DRAP-led task force and pilot access programs in tertiary hospitals in Pakistan. These views are aligned with the WHO Global Health Sector Strategy on Viral Hepatitis (2016-2030), which urges nations to implement new regulatory measures in order to increase access to life-saving therapies [21].

Another aspect that clinicians emphasized was the dire effects of inaction. In the absence of specific interventions, they predicted an increase in the levels of cirrhosis, hepatocellular carcinoma, and liver failure, as well as an increase in the requirements of liver transplantation and ICU attention. These issues reflect the global evidence that HDV infection worsens the disease rapidly and leads to mortality more than mono-infection with HBV [1]. Subsequently, respondents emphasized the social and economic effects of untreated HDV, such as lower quality of life, work incapacity, and expanding disparities in access to healthcare.

Finally, participants pointed to both international and local precedents that could inform Pakistan’s policy response. Internationally, conditional approval frameworks, compassionate access programs, and real-world evidence models have proven effective in accelerating treatment access for rare and high-risk diseases [22]. Locally, the Punjab Hepatitis Program demonstrates the feasibility of integrated, decentralized care and public-private partnerships in viral hepatitis control. These models, combined with the WHO’s Health Emergencies Programme and UNAIDS’ rights-based approaches, offer adaptable strategies for HDV.

Taken together, our findings highlight three key messages. First, diagnosis of HDV in Pakistan remains fragmented: although three-quarters of respondents routinely perform anti-HDV screening, only half can reliably access RNA PCR, primarily due to cost and availability constraints. Second, treatment access is perceived as highly inadequate, with clinicians emphasizing the absence of funded national programs and the need for structured pathways to access emerging HDV therapies. Third, policy and EUA mechanisms are viewed as essential levers; there is strong support for integrating HDV into the National Hepatitis Program, adopting national screening guidelines, and establishing EUA-style frameworks and pilot access programs under DRAP oversight.

Strengths and limitations

This exploratory mixed-methods study has several strengths, including the use of an adapted international HDV survey instrument, participation of clinicians from multiple major cities, and the integration of both quantitative and qualitative data to generate policy-relevant insights. However, important limitations must be acknowledged. The sample size was small (N=12), limiting statistical power and generalizability, and all participants were based in major urban tertiary centers, introducing an urban-centric bias that excludes perspectives from rural or primary-care settings. Diagnostic and treatment practices were self-reported and may not fully reflect actual clinical behavior, and estimates of co-infection rates or barriers may be influenced by recall or social desirability bias. Additionally, the study is clinician-centric and does not incorporate perspectives from patients, regulators, or broader health-system stakeholders, nor does it include cost-effectiveness or feasibility analyses. Taken together, these factors indicate that the findings should be interpreted as expert opinion and hypothesis-generating rather than as nationally representative estimates.

## Conclusions

This study highlights critical gaps in the diagnosis and management of HDV in Pakistan. Clinicians reported frequent encounters with HDV co-infection but emphasized limited and unaffordable diagnostic options, the absence of national guidelines, and a lack of awareness. Based on their consensus, the most urgent priorities include establishing national HDV screening guidelines for all HBsAg-positive patients, subsidizing and expanding access to HDV RNA testing to ensure affordability, and integrating HDV into the National Hepatitis Program to create coordinated pathways for diagnosis, treatment, and monitoring. Clinicians also supported early access to emerging therapies through structured regulatory mechanisms such as Emergency Use Authorization, drawing on successful international and local models. Urgent action across these areas is needed to prevent worsening disease burden, reduce inequities, and align Pakistan with global hepatitis elimination goals.

## Appendices

Annexure “A”

Hepatitis Delta Virus (HDV) Control & Awareness - KOL Questionnaire

Section 1: General Information

1. Designation:

2. City and Province of Practice:

3. Years of experience in Hepatology/Infectious Diseases:

Section 2: Clinical Observations & Disease Burden

6. Based on your practice, how frequently do you encounter HDV-positive patients?

☐ Rarely

☐ Occasionally

☐ Frequently

☐ Very Frequently

7. What percentage of your Hepatitis B patients are co-infected with HDV (approximate)?

☐ Less than 5%

☐ 5-10%

☐ 10-20%

☐ Over 20%

8. What challenges do you face in diagnosing HDV in your patients? (Check all that apply)

☐ Lack of awareness

☐ Limited testing availability

☐ High cost of testing

☐ Lack of national screening guidelines

☐ Others

9. Only answer this if you selected Others above (please specify)

(Open text response)

10. How often do you perform the following tests (Select for both)

a. HDV IgG Antibody

☐ Rarely Performed

☐ Occasionally Performed

☐ Routinely Performed

☐ Not Available

b. HDV RNA (PCR)

☐ Rarely Performed

☐ Occasionally Performed

☐ Routinely Performed

☐ Not Available

11. In your opinion, how affordable are HDV diagnostic tests (IgG and RNA) for patients in your practice setting? (Select one option) ☐ Affordable and widely accessible ☐ Available but not affordable for most patients ☐ Rarely available due to high cost ☐ Not available at all in our facility

Section 3: Disease Severity & Public Health Risk

12. In your clinical opinion, how dangerous or life-threatening is Hepatitis Delta compared to other forms of viral hepatitis?

☐ Mild

☐ Moderate

☐ Severe

☐ Critically Life-Threatening

13. What are the clinical consequences if Hepatitis Delta is not detected or treated in time? (Select all that apply)

☐ Rapid progression to cirrhosis

☐ Higher risk of liver failure

☐ Increased risk of hepatocellular carcinoma

☐ Poor response to existing HBV treatments

☐ Increased mortality

☐ Others

14. Only answer this if you selected Others above (please specify)

(Open text response)

15. If HDV is not addressed at a national level in the next 2-3 years, what public health challenges do you foresee?

(Open text response)

Section 4: Treatment Access & Infrastructure

16. Are there any approved or investigational HDV treatments available in your clinical setting?

☐ Yes

☐ No

17. If yes, please specify

(Open text response)

18. What are the key barriers in accessing HDV-specific treatment in Pakistan? (Check all that apply)

☐ Lack of drug registration

☐ Unaffordable cost

☐ No government procurement

☐ Absence of clinical treatment protocols

☐ Others

19. Only answer this if you selected Others above (please specify)

(Open text response)

20. In your opinion, how urgent is the need for a national-level HDV treatment access program?

☐ Not urgent

☐ Moderately urgent

☐ Very urgent

☐ Critical and immediate

Section 5: Government Policy & Recommendations

21. Do you believe HDV should be added as a separate component in the National Hepatitis Program?

☐ Yes

☐ No

☐ Not sure

22. Which of the following strategies do you recommend to control HDV in Pakistan? (Select top 3)

☐ National HDV screening guidelines

☐ Inclusion in existing Hepatitis B vaccination programs

☐ Awareness campaigns at the community and physician level

☐ Public-private partnerships to introduce HDV drugs

☐ HDV-specific training for doctors and nurses

☐ International collaboration for access to investigational drugs

23. What 3 key recommendations would you propose for the government to prioritize in combating HDV?

(Open text field)

Section 6: Final Thoughts

24. Would you be interested in participating in a focus group or policy roundtable to further discuss HDV strategy?

☐ Yes

☐ No

Section 7: Emergency Use Authorization (EUA) for HDV Treatment

25. Are you aware of any investigational or conditionally approved drugs currently under global development for the treatment of Hepatitis Delta Virus (HDV)?

☐ Yes

☐ No

☐ Aware to some extent

26. Only answer this if you selected “Aware to some extent” above (please specify)

(Open text response)

27. In your opinion, should Pakistan consider an Emergency Use Authorization (EUA) mechanism for HDV treatment if promising safety and efficacy data are observed during clinical trials conducted?

☐ Yes

☐ No

28. Under what conditions or safeguards should Pakistan allow emergency access to such investigational medicines for HDV? (Select Top three)

☐ Availability of Phase II/III interim data

☐ Drug is already approved in other countries or under compassionate use

☐ High unmet medical need with no local alternatives

☐ Oversight by national expert advisory board

☐ Patient consent and registry-based monitoring

☐ Others

29. Only answer this if you selected ' Others ' above (please specify).

(Open text field)

30. Do you think the Drug Regulatory Authority of Pakistan (DRAP) should have a special taskforce or framework to fast-track review of HDV-related investigational therapies?

☐ Yes

☐ No

☐ Only if endorsed by WHO or regional health authorities

31. Would you support a pilot treatment access program (via selected tertiary hospitals) to provide early access to HDV drugs before full registration, subject to data monitoring and ethics approval?

☐ Yes

☐ No

☐ Only with government oversight and sponsor responsibility

32. Please share any precedents or models (local or international) that Pakistan can follow to implement emergency access programs for rare or high-risk diseases like HDV.

(Open text field)
